# Water stress in *Musa* spp.: A systematic review

**DOI:** 10.1371/journal.pone.0208052

**Published:** 2018-12-03

**Authors:** Adriadna Souza Santos, Edson Perito Amorim, Claudia Fortes Ferreira, Carlos Priminho Pirovani

**Affiliations:** 1 Department of Biological Sciences, State University of Santa Cruz (UESC), Ilhéus, Bahia, Brazil; 2 Embrapa Mandioca e Fruticultura, Cruz das Almas, Bahia, Brazil; ICAR-Indian Institute of Agricultural Biotechnology, INDIA

## Abstract

**Background:**

The cultivation of bananas and other plants is limited by environmental stresses caused by climate change. In order to recognize physiological, biochemical and molecular components indicated to confer tolerance to water stress in *Musa* spp. we present the first systematic review on the topic.

**Methods:**

A systematic literature review was conducted using four databases for academic research (Google Academic, Springer, CAPES Journal Portal and PubMed Central). In order to avoid publication bias, a previously established protocol and inclusion and exclusion criteria were used.

**Results:**

The drought tolerance response is genotype-dependent, therefore the most studied varieties are constituted by the “B” genome. Tolerant plants are capable of super-expressing genes related to reisistance and defense response, maintaining the osmotic equilibrium and elimination of free radicals. Furthermore, they have higher amounts of water content, chlorophyll levels, stomatic conductance and dry root matter, when compared to susceptible plants.

**Conclusions:**

In recent years, few integrated studies on the effects of water stress on bananas have been carried out and none related to flood stress. Therefore, we highlight the need for new studies on the mechanisms of differentially expressed proteins in response to stress regulation, post-translational mechanisms and epigenetic inheritance in bananas.

## Introduction

Bananas are among the oldest cultivated plants to be domesticated [1]. They are classified botanically as monocotyledons of the Musaceae family which include the genus Ensete of Asian and African origin, the genus *Musella*, genetically close to Asian origin, and the *Musa* of East Asia, which is divided into sections with 22 (*Eumusa*, Rhodochlamys) and 20 chromosomes (*Australimusa*, Callimusa) [2]. Most edible bananas belong to the *Eumusa* section and are hybrids from *Musa acuminata* (genome "A") or from crosses with *Musa balbisiana* (genome "B"). A smaller group, including "Fe'i" or "Fehi" bananas, is limited to the Pacific region and is derived from *Australimusa* species [3].

The cross between species and subspecies of *M*. *acuminata* and *M*. *balbisiana* contributed to plant sterility and the appearance of parthenocarpy in the triploid and tetraploid genotypes [4].

It is known that the varieties constituted with the genome "A" produce fruits of high yield and quality, with long fingers and durability in the green and mature phases [5]. Molecular studies indicate that the "A" genome harbors more genes that are important for banana production and quality and can be used as candidates in breeding programs [6, 5]. However, bananas constituted with the "B" genome were domesticated under more severe climatic conditions, such as wide temperature variation and soil water scarcity, supporting better environmental stresses [7]

Plants undergo successive abiotic stress events during its life cycle. When water is not available to roots or when the transpiration becomes intense it is said that the plant is under water stress. Water stress may be caused by water deficit or high salinity in the soil. In the case of high salinity in the soil, periods of inundation and low soil temperature, there is still water in the solution of the soil, however, the plants are not capable of absorbing it, leading to a phenomenon called “physiological drought”[8].

Water deficit is one of the main limiting factors for the cultivation of *Musa* spp. In the highlands of East Africa, when annual rainfall is less than 1,100 mm, there may be losses of 20–60% in yield compared to other, more humid areas of the region. The weight of the bunch is affected due to the effect of water deficit during the flowering period, which reduces the number of fingers produced [9].

The response of banana varieties during periods of drought can be genotype-dependent [1]. Studies indicate that the presence of the "B" genome contributes to a better drought tolerance [1, 10]. However, in the absence of a conclusive definition of drought, it is a challenge to identify correct parameters and stress intensities for assessing water deficit tolerance [11].

This paper contributes to this challenge, since through a systematic review of the studies carried out over the past 10 years on the effects of water deficit on bananas and with the genetic sequencing of the species already carried out [12], it is possible to recognize, gather, classify and identify relevant new knowledge produced by other researchers.

A systematic review of the literature is a formalized and repetitive process where the state of the art of a given subject is documented through systematized searches in specific databases. This type of review is very common in Medical Sciences, since it is able to gather in a single document detailed and high level conclusions about diseases that one wishes to study [13, 14].

This paper proposes the first systematic review on water deficit in bananas. To guarantee its efficiency, the search process was conducted around a general objective, which in this review, was to recognize physiological, biochemical and molecular components indicated to confer drought tolerance on *Musa* spp.

## Materials and methods

The free software StArt (State of the Art through Systematic Review) v.3.3 Beta 03 was used to perform the systematic review. Developed by the Federal University of São Carlos (UFSCar), the tool provides computational support to researchers who seek answers to research questions using the systematic review technique. The software is available for download at the link: <http://lapes.dc.ufscar.br/tools/start_tool>.

**Fig 1:**
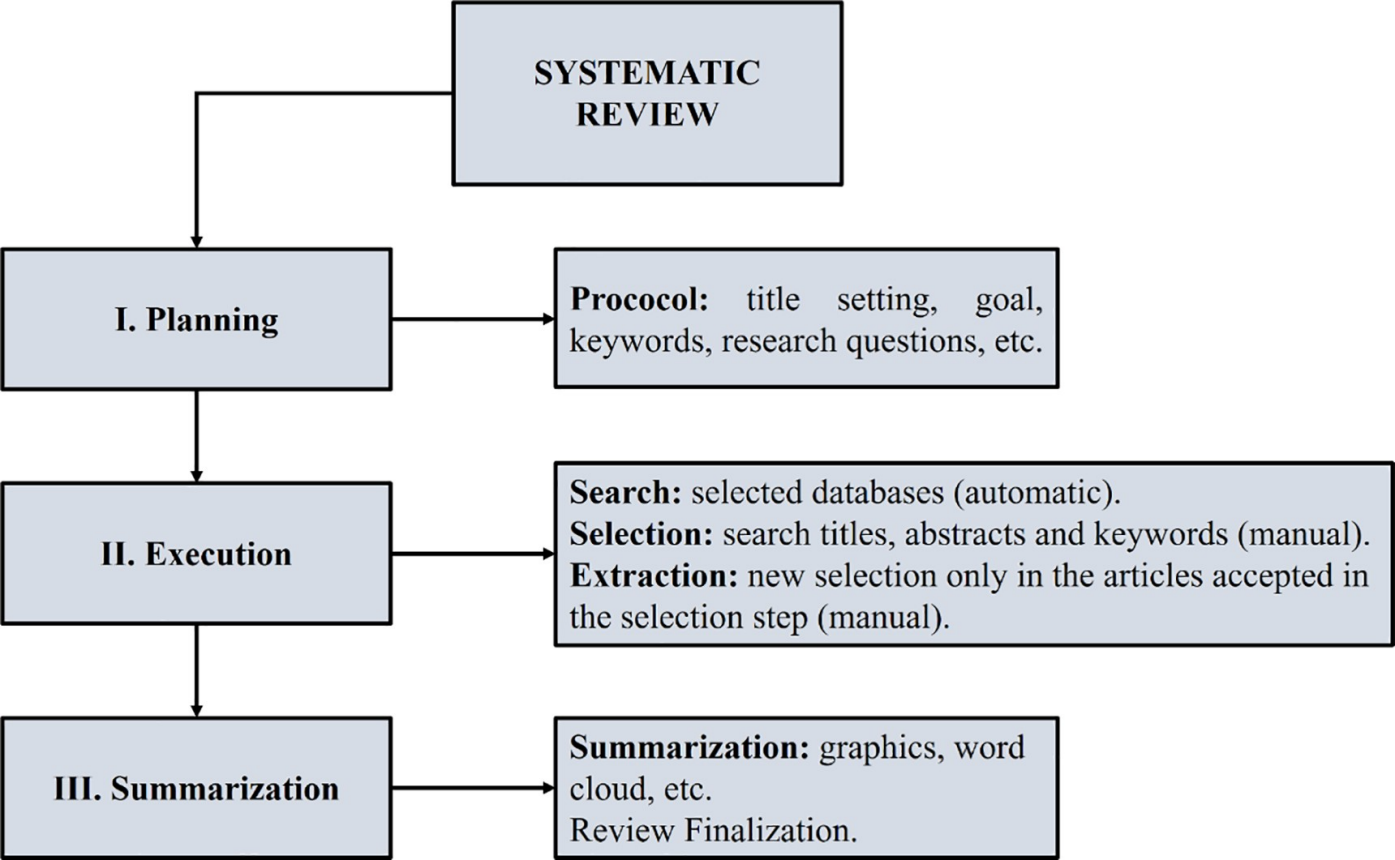
General systematic literature review flowchart.

The review process was run on StArt in three steps: Planning, Execution and Summarization (Fig 1).

Each step performed is described below:

I. Planning: A protocol was developed to be followed throughout the review process (http://doi.org/10.5281/zenodo.1465055). Title, objective, keywords, research questions, research sources, inclusion/exclusion criteria and the quality of the collected files were defined. The issues underlying this review are given in Table 1.

**Table 1:** List of review questions.

Research Questions
Q1. In what countries has more knowledge been produced about water stress in bananas?
Q2. What are the main institutions and/or groups involved in the study of water deficit tolerance?
Q3. What are the main genotypes and varieties studied?
Q4. What types of trials are proposed for studies of water stress?
Q5. What are the types of stresses addressed in papers on water stress?
Q6. Has there been any mention of using the banana genome?
Q7. Which components confer drought tolerance on *Musa* spp.?
Q8. What are the stressors used in the drought studies?

II. Execution: the searches were carried out in databases previously selected: Google Academic, Springer, CAPES Journal Portal and PubMed Central. The results were imported into BIBTEX, MEDILINE, RIS or Cochrane formats, compatible with StArt. The automatic search performed in the databases searches the central themes in the titles, abstracts and keywords. Relevant papers not found by searches were added later. To make the query expressive, the OR logical connector was used to group the synonymous keywords and AND to group the main parts. Thus, the search string used in all databases is represented in the following Box 1:

**Box 1: Search string**

("Musa" **OR** "banana") **AND** ("drought stress" **OR** "water deficit" **OR** "water stress")

In order to guarantee the international spectrum of the papers, only works written in English and available in academic channels were selected.

After automatic sorting, the manual selection and extraction phases were performed. In these phases, the inclusion and exclusion criteria are presented in the Table 2.

**Table 2:** Criteria used to include or exclude papers in the review process.

Inclusion Criteria	Exclusion Criteria
Papers that contain in the title, abstract or keywords, the terms banana (or *Musa*) and drought stress (or water deficit/stress), as detailed below in the search string.	Theses, dissertations, manuals and reports.
	Review Papers.
	Papers published in journals with no impact factor (individually checked on the sites: https://sucupira.capes.gov.br and https://www.scimagojr.com)*
	Papers without clear contribution.
	Papers published before 2008.

* Only one exception for the only article found on epigenetic studies in banana.

In the Selection, all papers imported to the software were classified as inclusion criteria: accepting, rejecting or excluding because they were duplicated. In Extraction, a new choice was made, considering only the papers accepted in the selection stage. In extraction, in addition to accepting, we can also reject and/or delete duplicate papers. PRISMA Checklist is presented in S2 Table.

III. Summarization: Graphs, Table and Word Cloud were generated to make up the systematic review.

## Results

### Search in databases

**Fig 2:**
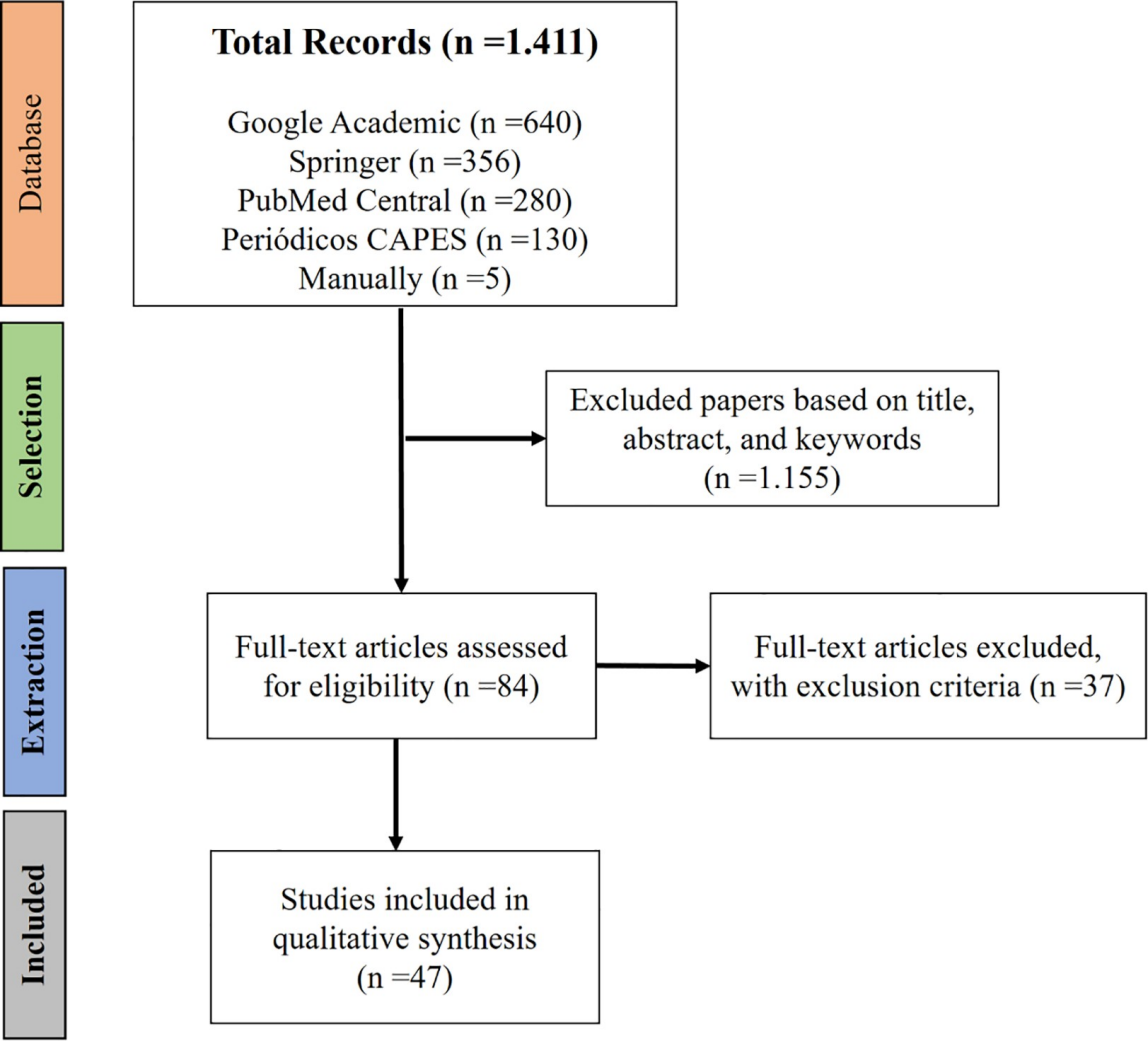
PRISMA flow diagram. Papers collected considering the search string in the databases.

The StArt software selected 1,410 papers related to the search string. No papers were found on water stress due to flooding in bananas. Google Academic contributed with the largest number of papers for this review, 46% of the total (Fig 2A). The majority of the papers in this database not related to the theme, gray literature (theses, dissertations, reports, annals …) and/or duplication, due to small differences in titles, such as capital letters, italicized formatting and accentuation. The Springer, PubMed Central and CAPES journals represented, respectively, 25%, 20% and 9% of the papers found, and presented lower rejection criteria (Fig 2).

Other databases could have been used, including Musalit (http://www.musalit.org/), which is specific to bananas. However, when using the search string, the results were restricted or insufficient, so we opted for databases with broader search spectrum such as Google Academic.

After Selection phase, only 6% or 84 papers out of a total of 1, 411 were accepted. In the Extraction phase, of the 84 previously selected papers, 55% were accepted, 45% were rejected by the exclusion criteria. Thus, we followed the review with 47 papers. For consultation purposes, the manuscripts were stored in a free access digital library at the following link: http://doi.org/10.5281/zenodo.1454052.

**Fig 3:**
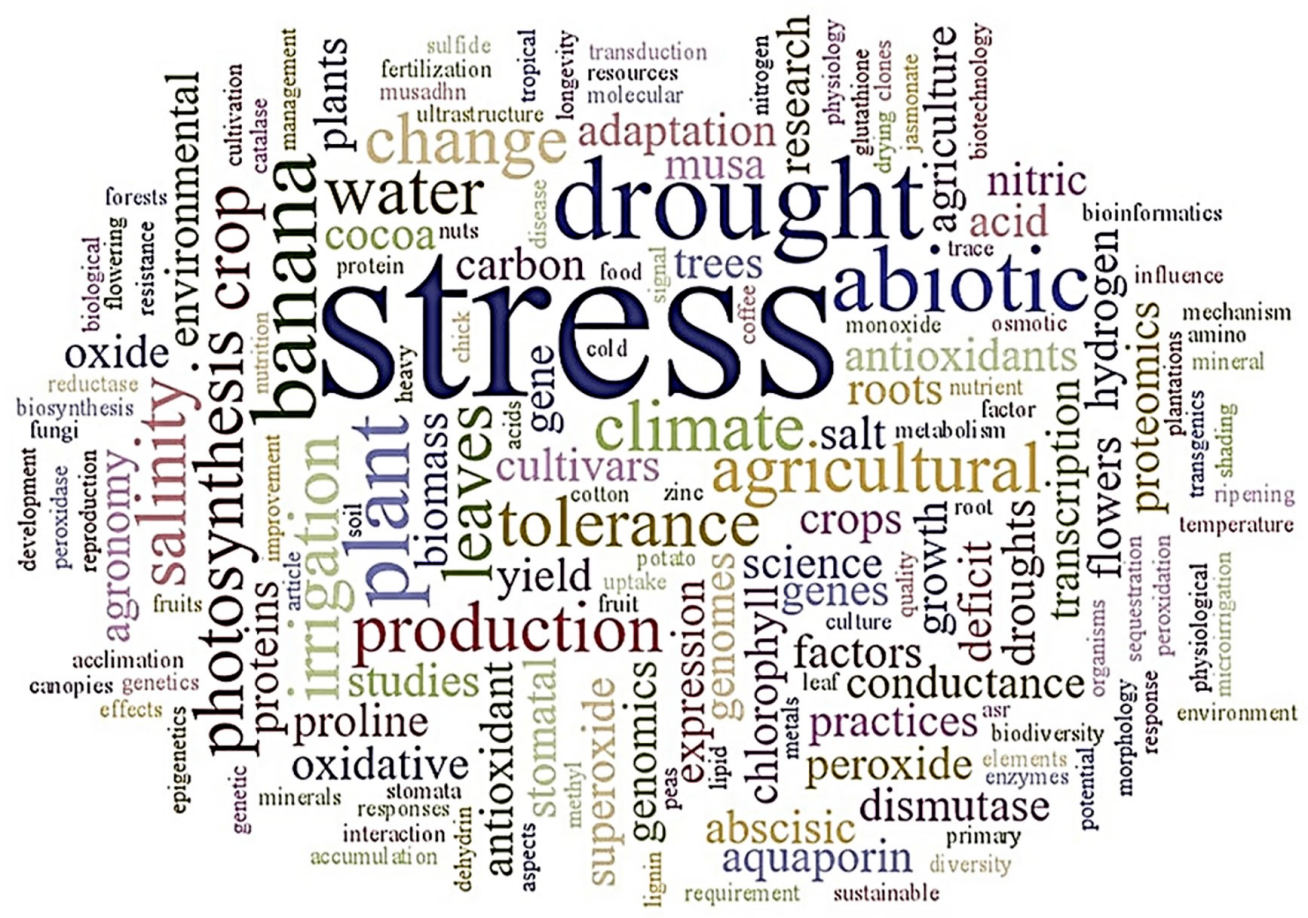
Relevant search word cloud.

A cloud of words was generated considering the frequency with which they appeared in papers of the Extraction phase (Fig 3). All the keywords used in database searches are represented and the other words are related to the research on water stress in banana plants.

### Research questions

**Fig 4:**
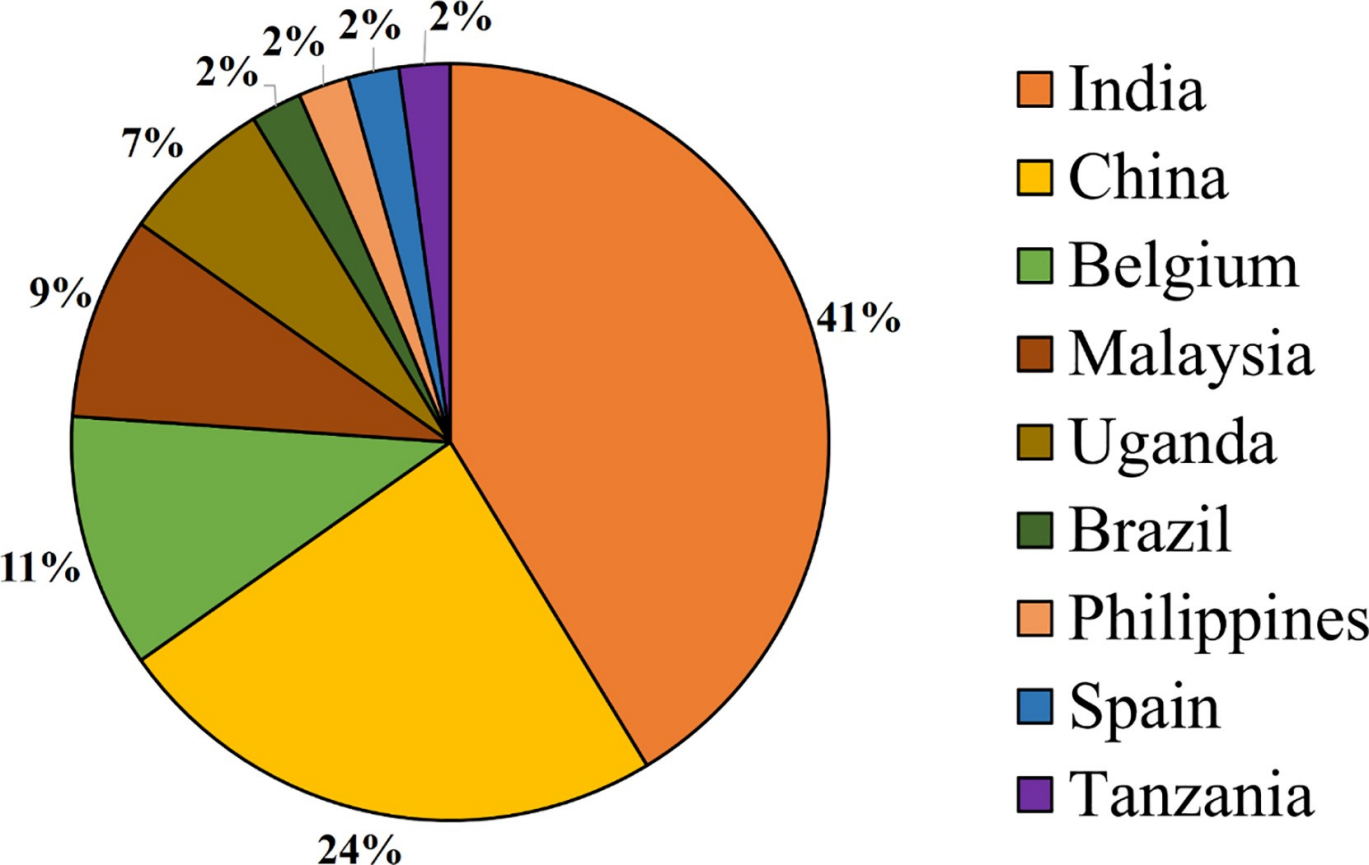
Main producing knowledge countries on water stress in banana plantations.

Most of the research works were developed in Asian countries, approximately 80% of the total of 47 (Fig 4). In Asia are also the main institutes and/or research institutions that are dedicated to the studies of Musa spp. under water deficit (S1 Table). In Europe, Africa and South America, 13%, 8% and 3% of the works were performed, respectively.

**Fig 5:**
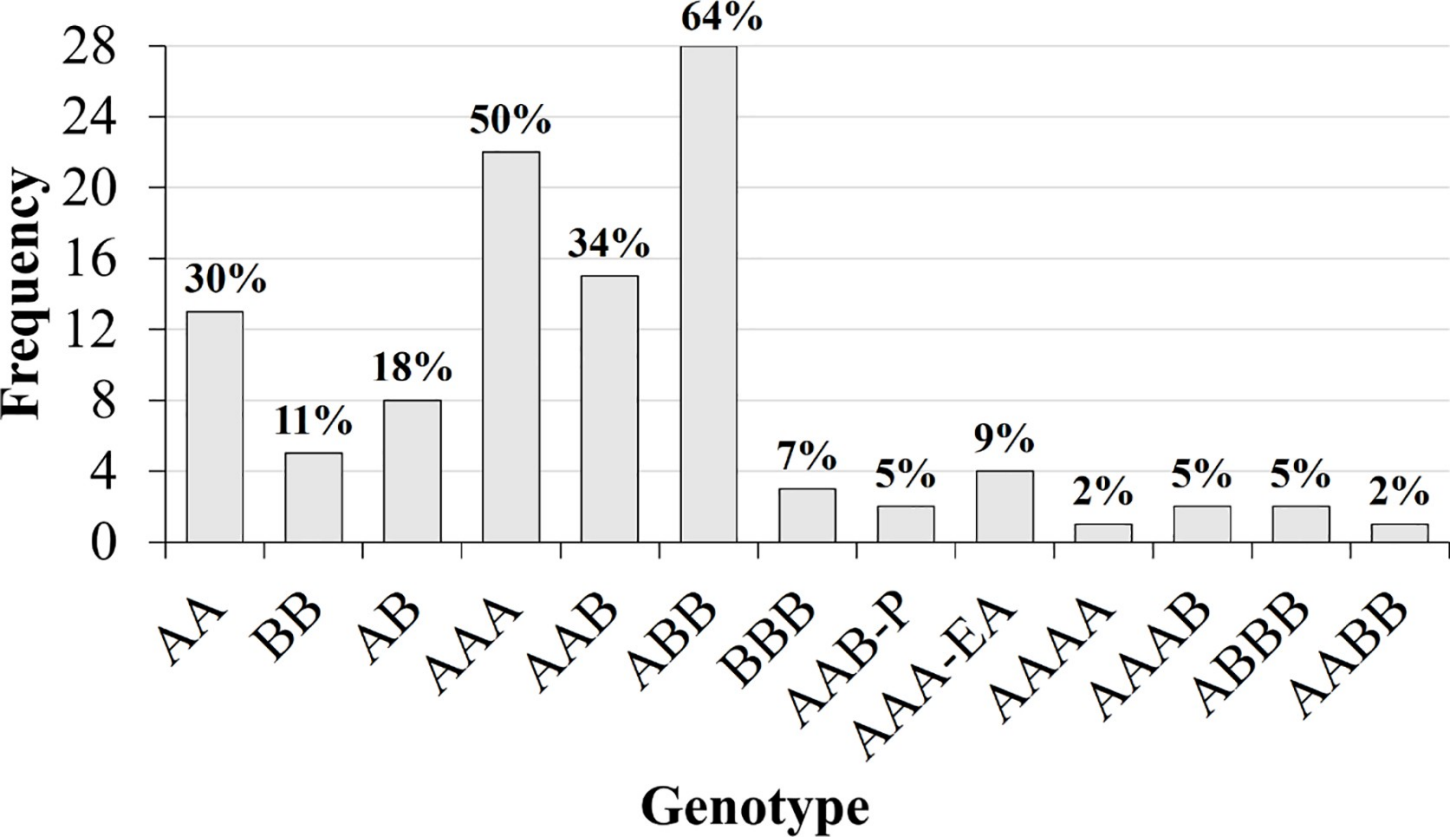
Frequency of genotypes of *Musa* spp. used in papers published in the last 10 years.

In the studies on water deficit, the main varieties studied are triploids AAA, AAB, ABB present in 50%, 34% and 64% of the papers, respectively (Fig 5). The diploids were studied in 59% of the works and the tetraploids in 14%. The total frequency of genotypes does not reflect the total number of papers in Fig 5, since some authors considered more than one genotype.

A list of cultivars and species used in the 47 papers is presented in Table 3. Among the most widely used varieties are triploids 'Saba', 'BaXi Jiao', 'Fen Jiao', 'Poovan', 'Karibale Monthan', 'Berangan', 'Cachaco' and 'Grande Naine', and the diploid 'Ney Poovan'.

**Table 3:** *Musa* spp. most used in studies on water deficit in the last 10 years.

Genomic Group	Genotypes (varieties/types)
**AA**	*M*. *acuminata*, *M*. *paradisiaca*, Matti, Sanna Chenkathali, Anaikomban, Calcutta-4.
**BB**	Bee hee kela, Bhimaithia.
**AB**	Ney Poovan.
**BBB**	Gubao, Cardaba, Saba Puti.
**AAA**	Berangan, Berangan Intan, Tianbaojiao, BaXi Jiao, Grande Naine, Brazilian, Yangambi Km5, Mpologoma, Mbwazirume, Williams, Uganda.
**AAA-EA**	Kisansa, Mbwazirume.
**AAB**	Rasthali, Latundan, PK Malaccacina, Pokpok, Sukali Ndizi, Popoulou, Nendran, Poovan, Karpuravalli.
**AAB-P**	Obino l'Ewai
**ABB**	Cachaco, Fen Jiao, Prata anã, Saba, Karibale Monthan, K. Namwa, Maduranga, Matavia, Paa Dalaga, Pelipia, Tindok, Kayinja, Karpooravalli.
**AAAB**	BRS Tropical.
**ABBB**	Tiparot.

**Fig 6:**
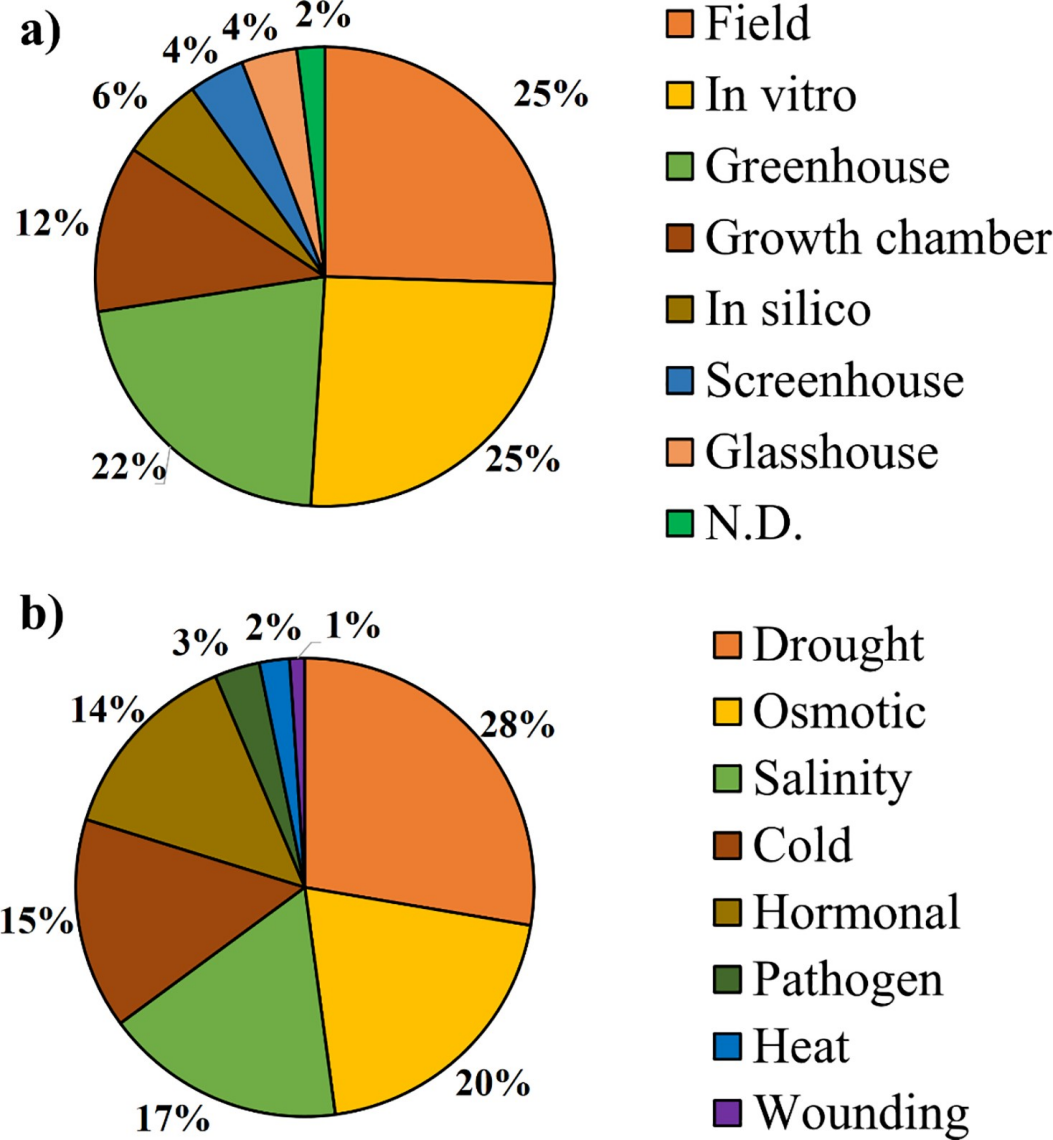
a) Frequency of study environments for application of water stress in banana plants. b) Types of stress applied on *Musa* spp.

Most of the studies were performed in the field, in vitro, greenhouse and growth chamber, corresponding to 30%, 30%, 25% and 14%, respectively. The remaining studies accounted for 19% (Fig 6A). The main stresses observed were drought, osmotic, salinity, cold and hormonal stress (Fig 6B). The following discussions considered only osmotic stress and water deficit, although some papers addressed multiple stresses.

Stress applied on Musa spp. were grouped into physical, chemical and biotic (Table 4). The agents causing water stress in selected papers were water deficit, water deficit progressive, PEG6000, mannitol, mannitol+PEG, sucrose+sorbitol and methyl viologen (Table 4).

**Table 4:** Classification of stresses applied on *Musa* spp.

Type of stress	Stressors
**Physical**	Water deficit, water deficit progressive, low temperature, high temperature, injury, NaCl.
**Chemical**	PEG6000, mannitol, mannitol+PEG, sucrose+sorbitol, methyl viologen, ethephon, ABA, AIA, MeJA, GI_3_, SA, CuSO_4_.
**Biotic**	*Fusarium oxysporum* f. sp. cubense, *Xanthomonas campestris* pv. musacearum (Xcm).

**Fig 7:**
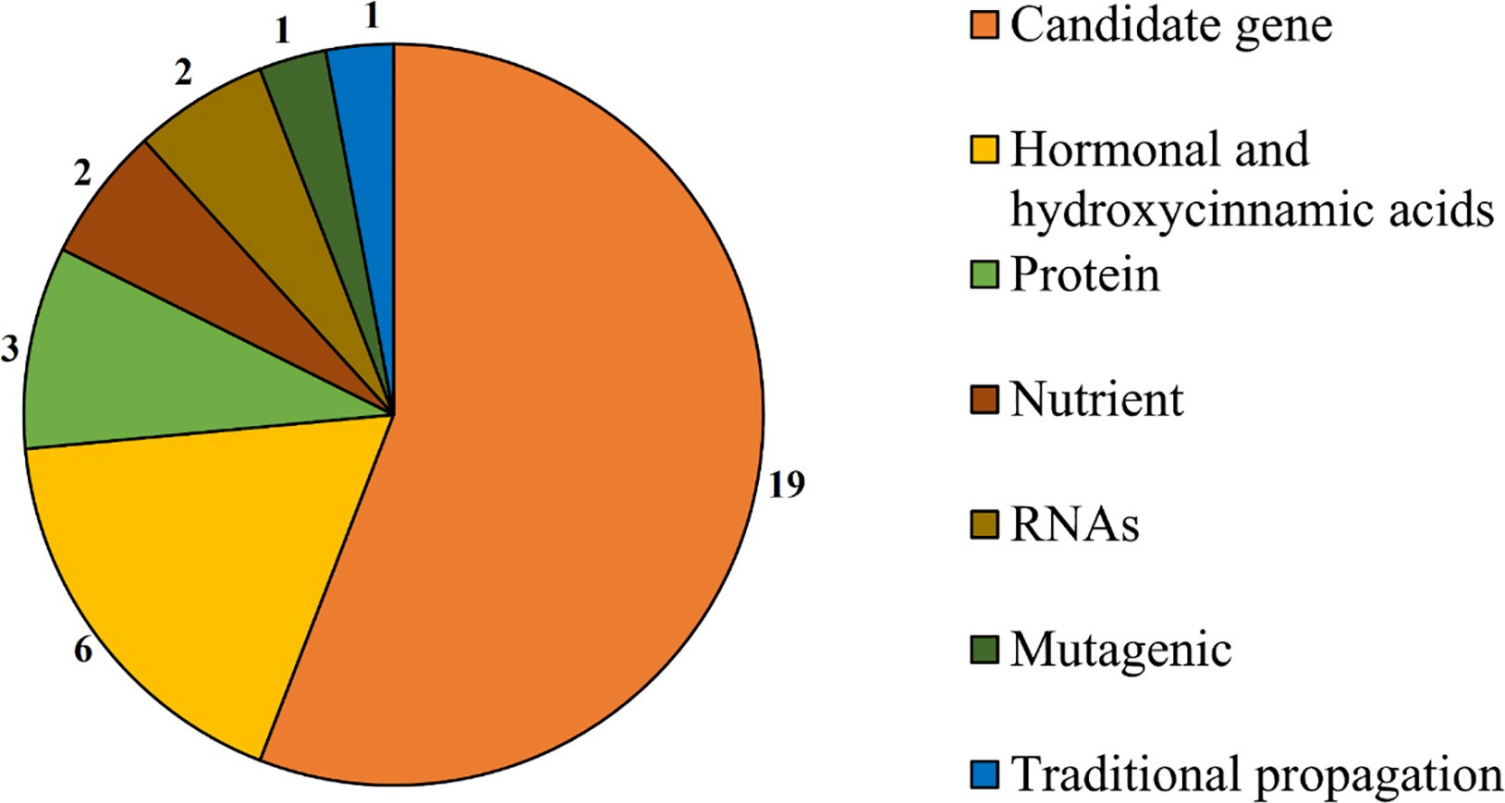
A summary of markers used in banana research.

Some markers have been successfully used in drought tolerance research in banana plantations with different applications (Fig 7). There are 21 candidate genes, 6 hormones, 3 proteins, 2 nutrients, 2 RNAs and 1 mutagenic agent.

Molecular markers and their effects under drought tolerance in *Musa* spp. are addressed in 21 papers (Table 5).

**Table 5:** Candidate gene used in research with *Musa* spp. and applications.

Candidate Gene	Applications	Authors
*AhSIPR10*	Improvement of photosynthetic efficiency and reduction of plasma membrane damage in the presence of NaCl and mannitol.	Rustagi et al., 2014 [15]
*MaAGPase*	Regulates the signaling pathway of biotic and abiotic stress and is involved in the development and maturation of fruits.	Miao et al., 2017 [6]
*MaAQP*	Promotes the early development of fruits, accelerating post-harvest banana maturation processes and plant resistance to saline and osmotic stress.	Hu et al., 2015 [16]
*MaARFs*	Involved in banana growth, fruit development, post-harvest maturation and responses to osmotic, saline and cold stress.	Hu et al., 2015 [17]
*MabZIP*	Involved in stages of organ development, fruit maturation and responses to abiotic stresses, including dry, cold and salt.	Hu et al., 2016 [5]
*MaCCS*	Induced in response to light, heat, drought stress, abscisic acid and indole-3-acetic acid.	Feng et al., 2016 [18]
*MaHsfs*	Involved in the growth of specific tissues or stages of development, such as fruit maturation and biotic and abiotic stress.	Wei et al., 2016 [19]
*MaPIP1;1*	It imparts tolerance to saline and water stress, reducing membrane damage, improving ionic distribution (K^+^/Na^+^ ratio) and maintaining the osmotic balance.	Xu et al., 2014 [20]
*MaSODs*	Plays an important role in the elimination of reactive oxygen species caused by abiotic and hormonal stresses in banana.	Feng et al., 2015 [21]
*MpASR*	Demonstrates positive activity to *F*. *oxysporum* f. sp. cubense and cold stress, dehydration, ABA and high salt concentration.	Liu et al., 2010 [22]
*MusaDHN-1*	Induced in leaves by drought, salinity, cold, oxidation, heavy metals, as well as by treatment with signaling molecules such as abscisic acid, ethylene and methyl jasmonate.	Shekhawat et al., 2011 [23]
*MusaNAC042*	Modulates the response to abiotic stress in banana preserving high levels of total chlorophyll and maintaining lower MDA content (malondialdehyde).	Tak et al., 2017 [24]
*MusaNAC68*	Regulates stress tolerance induced by NaCl and mannitol and root development.	Negi et al., 2015[25]
*MusaPIP1;2*	It improves survival characteristics under abiotic stress by maintaining low levels of malondialdehyde and high concentrations of proline, relative water content and photosynthetic efficiency.	Sreedharan et al., 2013 [26]
*MusaSAP1*	Involved in reducing malondialdehyde levels and regulating polyphenoloxidases (PPOs) that play important roles in multiple defense pathways.	Sreedharan et al., 2012 [27]
*MusaWRKY71*	An important constituent in the transcriptional reprogramming involved in several responses to stress in bananas, such as improved photosynthetic efficiency and reduction in leaf membrane damage.	Shekhawat et al., 2013 [28]
*Non-redundant* *DEGs*	Involved mainly in protein modifications, lipid metabolism, alkaloid biosynthesis, carbohydrate degradation, glycan metabolism, amino acid biosynthesis, cofactor, sugar-nucleotide, hormone, terpenoids and other secondary metabolites.	Muthusamy et at., 2016 [29]
*PYL-PP2C-SnRK2*	Regulates maturation and tolerance of banana fruits to cold, saline and osmotic stresses.	Hu et al., 2017 [8]
*MaSWEETs*	Increases sugar transport during initial fruit development and under abiotic and biotic stresses.	Miao et al., 2017 [30]
*WRKY*	Regulated in multiple stresses, involved in the growth, development and process of ripening fruits	Goel et al., 2016 [31]

Some papers have addressed the existence of ARNs regulating the response to water deficit in banana (Table 6).

**Table 6:** ARNs involved in drought tolerance in *Musa* spp.

RNAs	Applications	Authors
microRNAs	The *miR169*, *miR156* and *miR2118* were upregulated during stress due to water deficiency of the soil. MiR169 can control the expression of *NFY* and eventually the expression of *AQN* and *DHN* by reducing transcription, or by a mechanism of post-transcriptional degradation.	Muthusamy et al., 2014 [32]
LncRNAs	They are crucial regulators of gene transcription in plants in response to biotic and abiotic stress.	Muthusamy et al., 2015 [33]

Only two papers of this review presented the proteomic study of *Musa* spp. The ATPase, Heat shock and Dehydrogenases, are among the main proteins observed in contrasting banana genotypes for tolerance to water deficit (Table 7).

**Table 7:** Important proteins in the response of banana plants to water stress.

Protein	Applications	Authors
ATPase	Provides the main driving force for many cellular processes such as osmoregulation, signal transduction and reaction metabolism.	Mattos-Moreira et al., 2018 [34]
Heat shock	Promotes balance between antioxidants (AOX) and ROS during water stress.	
Dehydrogenases involved in NAD/NADH homeostasis	Limitation of ROS production by the NADPH matrix produced by NADP-isocitrate dehydrogenase and the non-proton-pumping transhydrogenase activities	Vanhove et al., 2012 [35]

Three papers deal with the Hormonal and Hydroxycinnamic Acids study to confer drought tolerance on banana varieties (Table 8).

**Table 8:** Hormonal and Hydroxycinnamic Acids in bananas under water déficit and its applications.

Hormonal and Hydroxycinnamic Acids	Applications	Authors
Methyl jasmonate	Improves drought tolerance, since it moderates the effects of oxidative stress, leading to better plant performance, fresh weight and shoot proliferation rate.	Mahmood et al., 2012 [36]
Abscisic Acid	Trigger adaptation of the plant to drought, as well as reduction of stomatal conductance, photosynthetic rate, plant growth in height, circumference, number of leaves and area.	Mahouachi et a., 2014 [37]
Indole-3-acetic acid	It can alleviate leaf senescence, improve survival levels and maintain cell stretching.	
Cinnamic acids	They are photoprotectors, since they are involved in the folding mechanism of the leaves, which reduces the area of irradiation and loss of water.	
Ferulic acids		
Salicylic acid	They may be limited to a rapid and transient increase at the beginning of the first stress period, as they did not respond to consecutive stresses.	
Jasmonic acid		
Salicylic acid	Promotes increase in the rate of proliferation, fresh weight gain, maintenance of the relative water content and reduction of H_2_O_2_.	Bidabadi et al., 2012 [38]

**Fig 8:**
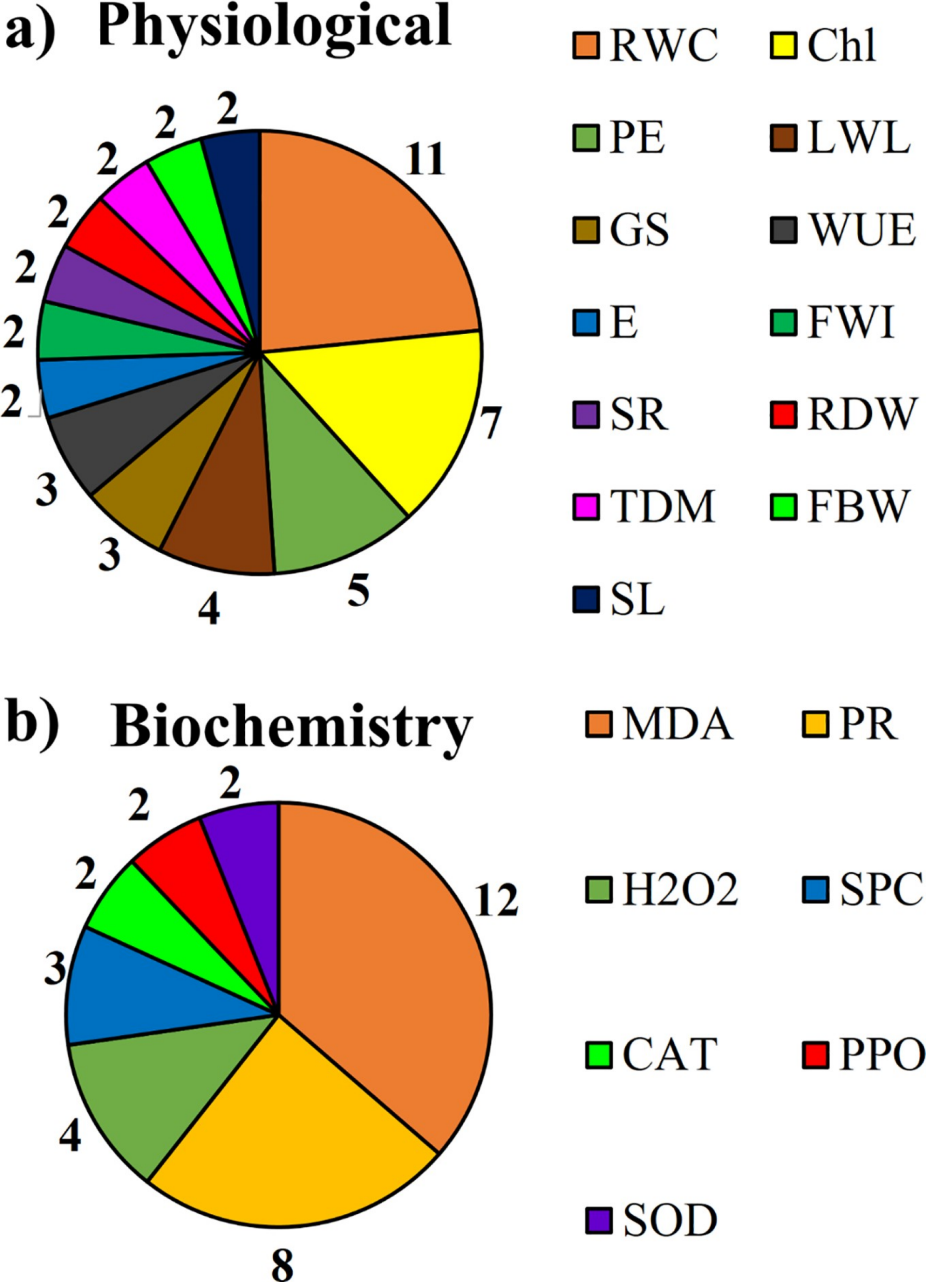
Main biochemical and physiological parameters measured. a) RWC—Relative water content, Chl—chlorophyll levels, PE—Photosynthetic efficiency, LWL—leaf water loss, GS—Stomatal conductance, WUE—Water Use Efficiency, E—Transpiration rate, FWI- fresh weight increase, SR- survival rates, RDW- Root dry weight, TDM- Total dry matter yield, FBW- fresh bunch weight, SL- Shoot Length. b) MDA—Malondialdehyde, PR- Proline, H_2_O_2_—Hydrogen peroxide, SPC—Soluble protein content, AN—Acid ninhydrin, CAT—Catalase, PPO—Polyphenol oxidase, SOD- Superoxide dismutase.

Relative water content, chlorophyll levels, photosynthetic efficiency and leaf water loss are among the main physiological analyzes used to measure of the effects of water stress on banana genotypes (Fig 8A). The content of Malondialdehyde, Proline, Hydrogen peroxide and Soluble protein are among the main biochemical analysis (Fig 8B)

## Discussion

This systematic review presentes a compilation of quality articles and main institutions involved in the study of tolerance to water stress in *Musa* spp. The great interest of Asian countries in the study of the water deficit in *Musa* sp. probably stems from the fact that these countries are at the center of origin and world banana trade. Molecular evidence confirmed that edible banana genotypes originated in the islands of Southeast Asia and developed through complex pathways of geodomestication [39]. India is by far the world's largest banana producer, and China is the fourth largest importer. Malaysia is also among the domestic market consumers in Asia. The Philippines was the world's second largest banana exporter by 2015, when they lost their position due to the long period of drought caused by El Niño [40]. Brazil is among the five largest producers in the world and the European Union and the United States are among the largest importers of this fruit, approximately 60% of the global distribution [40].

The triploid cultivars are the most studied. In natural germplasm, there are few ABB triploid varieties that have good palatability and high productivity, and usually the most consumed are the Cavendish (AAA) subgroup, such as Grande Naine and Williams [39, 41]. Therefore, an improvement strategy is to combine edible AB cultivars with wild *M*. *balbisiana* to create new ABB varieties that are productive, palatable and tolerant to drought and cold [1]. In nature, *M*. *acuminata* and its subspecies are typically delicate and thin, and require favorable environmental conditions for good development, since they originated in the wetlands of Asia. *M*. *balbisiana* was diversified and domesticated in regions of drought-prone monsoons in Southeast Asia, which probably contributed to tolerance to abiotic stresses [9].

The most common growth environment were field and in vitro and the main stresses applied were water deficit and osmotic stresses. Drought stresses are given by the suspension of irrigation in plants grown in the greenhouse [39] or in typically dry seasons with commercial plantations [3]. Field and greenhouse studies generally approach the conditions of agricultural cultivation [4]. Mannitol, polyethylene glycol and methyl viologen are organic compounds with osmotic action. As liquid solution, they are commonly used to simulate water deficit conditions [5]. In the selected works, mannitol was used, for example, in a water stress test with five leaves of the cultivars 'BaXi Jiao' and 'Fen Jiao' [6, 10], in different leaf discs *Musa* spp. [5, 15, 25, 42] and in vitro in the variety ‘Karibale Monthan’ [27]. Polyethyleneglycol (PEG) was used in vitro as it has a high molecular weight and reduces the water potential of the medium, thus inhibiting the growth of bananas, which can not absorb water and nutrients [7, 9, 38]. The methyl viologen was used in leaves of transgenic banana plants to simulate osmotic stress, as it acts by receiving electrons from the primary acceptors of the photosystem-I and transferring them to the oxygen, generating ROS [43]. Sorbitol was considered a neutral inducer of osmotic stress in banana when compared to culture media that use sucrose as the only source of carbon [2]. Therefore, cultures initially acclimated with sucrose in vitro respond better to osmotic stress, but over time, sugar metabolism consumes ATP and boosts respiration, resulting in limiting levels of oxygen, which can eventually lead to anoxia. [10].

In all studies on osmotic stress, the efficiency of at least one of the compounds was determined in the selection of water stress tolerant genotypes. In vitro studies are considered the first step in characterizing the biodiversity of tolerant *Musa* spp. in a germplasm bank, since they allow a high yield and control of the experiment, however, the artificial conditions are pointed out as disadvantages [18].

More than 20 candidate genes were indicated as potential markers for banana drought tolerance. The *MaAGPase*, *MaAQP*, MabZIP, non-redundant DEGs, MaSWEETs and *PYL-PP2C-SnRK2* genes showed genome-dependent response in genome "B" compared to genome "A" [26, 30, 38, 44, 45]. The expression of cuticular wax biosynthesis genes (*FATB* and *KCS11*) is also a dependent genotype, influencing the water retention capacity of leaves and mitigating the effects of water stress on varieties constituted by the "B" genome, a promising line for future studies [46]. [38] suggests that the degradation of mRNA driven by the enzyme exoribonuclease 5'-3' during abiotic stress may be responsible for sensitivity to drought in cv. 'Grande Naine' (AAA).

In one study, the heterologous expression of the banana aquaporin gene, *MaPIP1;1*, in Arabidopsis was presented as an alternative to improve the response of species, independent of the genotype, since in Arabidopsis it showed an increase in the primary root length, number of root hairs and survival rates [47]. The same stress acclimation behavior was observed in transgenic plants overexpressing *MusaNAC68* [25].

The banana transcriptome and proteome are also altered in response to water stress. The expression of RNAs involved in drought tolerance of the the ‘Saba’ cultivar (“ABB” genome-tolerant) and Grande Naine (“AAA” genome-susceptible) depends on the genetic constitution of the cultivars, whereas the genome “B” is the most efficient [46, 48]. The proteome analysis of banana plants submitted to water stress shows that there is a new equilibrium in stressed plants and that respiration, ROS metabolism, growth and development, especially of plants with the "B" genome, are altered in order to adjust to tolerate stress [16, 18].

The exogenous use of Hormone and Hydroxycinnamic Acids to confer tolerance to *Musa* spp. triploids (AAA) seems promising. With the exception of Salicylic acid and Jasmonic acid that did not present a significant response in progressive water stress [20], all other acids contributed to the acclimatization of banana varieties to water deficit [21, 33].

In addition to exposure to hormones, water stress tolerant lineages can be obtained from variations induced by ethyl methanesulphonate (EMI) [34] or by maintaining the nitrogen and potassium ratio in the soil, since potassium mitigates the production of dry matter and loss of bunches resulting from water stress in the early stages of development [36].

Of the 47 articles in this review, only 13 cited the use of the banana genome. Until 2009, few complete studies on abiotic stress in bananas had been published, and none on drought [7]. The sequencing of the *Musa acuminata* genome, whose DNA is in the composition of all edible varieties [13], is an important step for the knowledge of genes with specific functions and of interest for the genetic improvement of the species.

The first epigenetic study relating to the response of in vitro micropropagated banana varieties to mechanisms underlying abiotic stresses was published in 2012. Cytosine hypomethylation of DNA is related to increased leaf stomata density, resulting in excessive water loss and increased foliar senescence [49]. This work was published in a journal with no impact factor. Their results are presented due to the importance of this knowledge for the production of varieties tolerant to water stress.

A series of physiological and biochemical changes were observed in different banana varieties submitted to water stress. In superior plants, a small part of the water absorbed by the roots is used in photosynthesis and biomass production and most of it is returned to the environment in the process and transpiration [49]. The stomatal closure is one of the main strategies used by plants to control the amount of water evaporated into the medium [11]. Under conditions of water deficit, the relative water content in the leaves decreases, consequently leading to the reduction of stomatal conductance, photosynthetic rate [50,51,52] and transpiration rate [53]. Within the cells, the formation of reactive oxygen species (ROS) occurs, leading to the formation of harmful free radicals, lipid peroxidation [47] and denaturing proteins [30]. Species susceptible to water stress are more vulnerable to increased polyphenol oxidase, which is an enzyme which catalyzes the darkening reaction that occurs in many fruits and vegetables [54]. Varieties of bananas tolerant to water deficit are capable of increasing the activity of osmoprotective components, such as Proline, which eliminate free radicals in the cytoplasm and increase the solubility of poorly soluble proteins, free amino acids and total sugars [55, 56]. The activity of antioxidant enzymes, such as Catalase, Superoxide dismutase and Peroxidase of ascorbate [57] is also higher in tolerant species when compared to those susceptible to water stress.

## Conclusions

Bananas are a product of export and subsistence in several countries. They are susceptible to a wide range of biotic and abiotic stresses where cultivars with the "B" genome show some superiority over those presenting the "A" genomes, such as increased expression of genes related to plant defense, higher relative content of water, length of primary roots and fresh bunch weight as well as better rates of survival and photosynthesis. Many efforts have been made in studies on the effect of water stress on the expression of genes that are candidates for drought tolerance, but few studies in the last 10 years have addressed the expression of proteins and post-translational mechanisms. We also highlight the need to study the epigenetic inheritance in bananas, elucidating the effects of environmental stresses on DNA and chromatin sequences and possible regulatory mechanisms that are maintained and passed on to the next generations, making them more tolerant through genetic “*imprinting”*.

## Supporting information

S1 Table Main institutions responsible for the production of worldwide agriculture knowledge. (DOCX)

S2 Table PRISMA checklist. (DOC)
