# Supplementary material for: Water stress in Musa spp.: A systematic review
Source: PLoS One. 2018 Dec 3;13(12):e0208052. doi: 10.1371/journal.pone.0208052 (PMC6277099; doi:10.1371/journal.pone.0208052)
Supplement: S1 Table — (DOCX) [file pone.0208052.s001.docx]

| **Frequency** | **Institutions** |
| --- | --- |
| 7 | Institute of Tropical Bioscience and Biotechnology/Chinese Academy of Tropical Agricultural Sciences. Website: < http://en.catas.cn/contents/1270/64347.html> |
| 6 | Regional Research Station/Tamil Nadu Agricultural University. Website: <http://www.tnau.ac.in/index.html > |
| 6 | Plant Cell Culture Technology Section, Nuclear Agriculture and Biotechnology Division, Bhabha Atomic Research Centre. Website: <http://www.barc.gov.in/index.html> |
| 5 | Indian Council of Agricultural Research/National Research Centre for Banana. Website: <http://nrcb.res.in/> |
| 3 | Katholieke Universiteit Leuven. Website: <https://www.kuleuven.be/kuleuven/> |
| 2 | International Institute of Tropical Agriculture. Website: <http://www.iita.org/> |
| 2 | Rwanda Agriculture Board. Website: <http://www.rab.gov.rw/home/> |
| 2 | University Putra Malaysia. Website: <http://www.upm.edu.my/> |
| 2 | Department of Horticulture, College of Agriculture, Isfahan University of Technology. Website: <https://agri.iut.ac.ir/en> |
| 2 | Institute of Horticultural Biotechnology/Fujian Agriculture and Forestry University. Website: <http://english.fafu.edu.cn/> |
| 2 | School of Life Sciences/Jawaharlal Nehru University- Sun Yat-Sen University. Website: <http://www.sysu.edu.cn/2012/en/index.htm > |
| 1 | Department of Biotechnology, University of Agricultural Sciences. Website: <http://www.uasbangalore.edu.in/> |
| 1 | Departamento de Ingeniería, Producción y Economía Agraria, Carretera de Geneto. Website: <https://www.ull.es/> |
| 1 | Bioversity International/National Agricultural Research Organization. Website: <https://www.naro.go.ug/> |
| 1 | College of Agriculture, Hainan University. Website: <http://www.hainu.edu.cn/guojiwenhua-en/> |
| 1 | Council of Scientific and Industrial Research-National Botanical Research Institute. Website: <http://www.nbri.res.in/> |
| 1 | [Universidade Estadual de Feira de Santana](http://www.uefs.br/)/Embrapa Mandioca e Fruticultura. Website: <https://www.embrapa.br/mandioca-e-fruticultura> |
| 1 | Crop Science Cluster-Institute of Plant Breeding, College of Agriculture, University of the Philippines Los Baños College. Website: < http://uplb.edu.ph/ > |
| 1 | Department of Crop Science and Production, Sokoine University of Agriculture. Website: < http://www.sua.ac.tz/ > |
