# Supplementary material for: Water stress in Musa spp.: A systematic review
Source: PLoS One. 2018 Dec 3;13(12):e0208052. doi: 10.1371/journal.pone.0208052 (PMC6277099; doi:10.1371/journal.pone.0208052)
Supplement: S2 Table — (DOC) [file pone.0208052.s002.doc]

| **Section/topic** | **#** | **Checklist item** | **Reported on page #** |
| --- | --- | --- | --- |
| **TITLE** | | |  |
| Title | 1 | **WATER STRESS IN *Musa* spp.: A SYSTEMATIC REVIEW** |  |
| **ABSTRACT** | | |  |
| Structured summary | 2 | The cultivation of bananas and other plants is limited by environmental stresses caused by climate change. In order to recognize physiological, biochemical and molecular components indicated to confer tolerance to water stress in Musa spp. we present the first systematic review on the topic. A systematic literature review was conducted using four databases for academic research (Google Academic, Springer, CAPES Journal Portal and PubMed Central). To avoid publication bias, a previously established protocol and inclusion and exclusion criteria were used. The results indicate that most studies on water stress were published in Asian countries. In addition, the drought tolerance response is dependent genotype, probably because of this, the most studied varieties are constituted by genome "B". The most used culture environments were the field and in vitro and the main stresses applied were water deficit and osmotic stresses. In recent years, few integrated studies on the effects of water stress on banana trees have been carried out and none have dealt with flood stress. Therefore, we highlight the need for new studies on the mechanisms of protein regulation differentially expressed in response to stress, post-translational mechanisms and epigenetic inheritance in banana plants. |  |
| **INTRODUCTION** | | |  |
| Rationale | 3 | The absence of a conclusive definition of drought, it is a challenge to identify correct parameters and stress intensities for the assessment of water deficit tolerance. This paper contributes to this challenge, since through a systematic review of the studies carried out over the past 10 years on the effects of water deficit on banana trees and the genetic sequencing of the species already carried out, it is possible to recognize, classify and identify new knowledge produced by other researchers. |  |
| Objectives | 4 | This paper proposes the first systematic review on water deficit in banana. To guarantee its efficiency, the search process was conducted around a protocol established and a general objective, which in this review was to recognize physiological, biochemical and molecular components indicated to confer drought tolerance on Musa spp. |  |
| **METHODS** | | |  |
| Protocol and registration | 5 | Relevant papers not found by searches were added later. To make the query expressive, the OR logical connector was used to group the synonymous keywords and AND to group the main parts. Thus, the search string used in all databases is represented in the following box:  ("muse" OR "banana") AND ("drought stress" OR "water deficit" OR "water stress").  In order to guarantee the international reach of the papers, only works written in English and available in academic channels were selected. |  |
| Eligibility criteria | 6 | Only papers published in English with a clear contribution on the theme were published, published in journals with impact factor, published in the last 10 years. Only five papers were added manually, since they were not found by the databases used. A pioneering article on epigenetics was added, although it was published in the journal without impact factor. |  |
| Information sources | 7 | The researches were carried out in databases previously selected: Google Academic, Springer, CAPES Journal Portal and PubMed Central. The results were imported into BIBTEX, MEDILINE, RIS or Cochrane formats, compatible with StArt. |  |
| Search | 8 | For google academic, for example, the search string was pasted into the search bar and all the results of the last 10 years were exported in BibTeX format to the software StArt. |  |
| Study selection | 9 | The automatic search performed in the databases searches the central themes in the titles, abstracts and keywords. |  |
| Data collection process | 10 | During the reading of the articles the following questions were answered:  Q1. In what countries has more knowledge been produced about water stress been produced in banana trees?  Q2. What are the main institutions and/or groups involved in the study of water deficit tolerance?  Q3. What are the main genotypes and varieties studied?  Q4. What types of trials are proposed for studies of water stress?  Q5. What are the types of stresses addressed in papers on water stress?  Q6. Has there been any mention of using the banana genome?  Q7. Which components confer drought tolerance on Musa spp.?  Q8. What are the stressors used in the drought studies? |  |
| Data items | 11 | Abiotic stresses, such as water deficit, osmotic and flood in banana. |  |
| Risk of bias in individual studies | 12 | The inclusion and exclusion criteria were followed during the selection of papers, except for epigenetic article due to the relevance of the information as a new alternative to study the tolerance of the water deficit in banana. |  |
| Summary measures | 13 | We did not use meta-analysis in the study |  |
| Synthesis of results | 14 | We did not use meta-analysis in the study. |  |

Page 1 of 2

| **Section/topic** | **#** | **Checklist item** | **Reported on page #** |
| --- | --- | --- | --- |
| Risk of bias across studies | 15 | We did not carry out risk assessment of bias that could affect the cumulative evidence, only following the pre-established criteria. |  |
| Additional analyses | 16 | No additional methods of analysis were used. |  |
| **RESULTS** | | |  |
| Study selection | 17 | StArt software has selected 1.410 articles related to the search string.  Google Academic contributed the most articles for this review, 46% of the total. The Springer, PubMed Central and CAPES journals represented, respectively, 25%, 20% and 9% of the articles found, and presented less rejection criteria. Only 6% or 84 articles, out of 1,410, were accepted. Of the 84 articles previously selected, 55% were accepted, 39% were rejected by the exclusion criteria and 6% were duplicates. Thus, we followed the review with 47 articles. |  |
| Study characteristics | 18 | The research questions were applied in all articles to extract the information that supports the review. |  |
| Risk of bias within studies | 19 | The only item that was selected outside the exclusion criteria was the following: MSOGOYA, T.J., GROUT B. W. (2012) Cytosine DNA methylation changes drought stress responses in tissue culture derived banana (Musa AAA – East Africa) plants. Journal of Applied Biosciences. 49: 3383– 3387. |  |
| Results of individual studies | 20 | For all outcomes considered (benefits or harms), present, for each study: (a) simple summary data for each intervention group (b) effect estimates and confidence intervals, ideally with a forest plot. This was the only article that addressed epigenetic inheritance. |  |
| Synthesis of results | 21 | We do not use meta-analysis. |  |
| Risk of bias across studies | 22 | We did not do risk assessment. |  |
| Additional analysis | 23 | No additional methods of analysis were used. |  |
| **DISCUSSION** | | |  |
| Summary of evidence | 24 | - The interest of the Asian countries in the study of the water deficit in Musa sp. Probably stems from the fact that these countries are in the center of origin and world banana trade.  - Triploid cultivars are the most studied because they fit the edible genotypes.  - The most used culture environments were field and in vitro. Field studies approach natural conditions and in vitro assays are faster and under controlled conditions.  - The most commonly used strategy to confer drought tolerance is the overexpression of candidate genes. |  |
| Limitations | 25 | - Despite the importance of banana cultivation for fruit growing, there are few epigenetic, proteomic and transcriptomic studies. |  |
| Conclusions | 26 | Bananas are a product of export and subsistence in several countries. They are susceptible to a wide range of biotic and abiotic stresses, where cultivars with the "B" genome show some superiority over those presening the "A" genomes. Many efforts have been undertaken in studies of the effect of water stress on expression of candidate genes for drought tolerance, but few studies over the last 10 years have addressed protein expression and post translational mechanisms. We also highlight the need to study epigenetic inheritance in bananas, elucidating the effects of environmental stresses on DNA sequences and chromatin and possible regulatory mechanisms that are maintained and passed on to next generations, making them more tolerant through genetic “*imprinting*”. |  |
| **FUNDING** | | |  |
| Funding | 27 | The CAPES-Embrapa Program funds research conducted during the Ph.D. |  |

*From:*  Moher D, Liberati A, Tetzlaff J, Altman DG, The PRISMA Group (2009). Preferred Reporting Items for Systematic Reviews and Meta-Analyses: The PRISMA Statement. PLoS Med 6(7): e1000097. doi:10.1371/journal.pmed1000097

For more information, visit: **www.prisma-statement.org**.

Page 2 of 2
